# Immunophenotypic Profile of Normal Hematopoietic Populations in Human Bone Marrow: Influence of Gender and Aging as a Basis for Reference Value Establishment

**DOI:** 10.3390/cells14171392

**Published:** 2025-09-06

**Authors:** Flavia Arandas de Sousa, Rodolfo Patussi Correa, Laiz Cameirão Bento, Luiz Fabiano Presente Taniguchi, Nydia Strachman Bacal, Luciana Cavalheiro Marti

**Affiliations:** 1 Laboratory of Clinical Pathology, Hospital Israelita Albert Einstein, São Paulo 05652-000, SP, Brazil; flavia.sousa@einstein.br (F.A.d.S.); rodolfoptc@gmail.com (R.P.C.); laiz.bento@einstein.br (L.C.B.); nydia.bacal@einstein.br (N.S.B.); 2 Orthopedic and Traumatology Department, Hospital Municipal Dr. Moysés Deutsch, São Paulo 04853-000, SP, Brazil; luiz.taniguchi@hmbm.org.br; 3 Experimental Research Center, Hospital Israelita Albert Einstein, São Paulo 05653-000, SP, Brazil

**Keywords:** bone marrow flow cytometry, normal values, hematopoietic cells, age-related variations, gender-related variations

## Abstract

The purpose of this study was to evaluate normal values of healthy human bone marrow (*n* = 56) and identify gender- and age-related variations using cell lineage markers and maturational curves. Using 10-color quantitative flow cytometry, various cell types were identified, including B cells, T cells, NK cells, granulocytes, monocytes, erythroblasts, plasma cells, basophils, mast cells, and dendritic cells. Results revealed significant age-related declines in the absolute counts of nucleated cells (*p* = 0.001), including CD34+ immature B cells (*p* = 0.006) and CD34- immature B cells (*p* = 0.004). Declines were also observed for T cells (*p* = 0.002), cytotoxic T cells (*p* < 0.001), double-negative T cells (*p* = 0.0001), NK cells (*p* = 0.007), CD16- NK cells (*p* < 0.001), metamyelocytes (*p* = 0.002), neutrophils (*p* = 0.001), basophils (*p* = 0.009), promonocytes (*p* = 0.001), mature monocytes (*p* = 0.007), and plasmacytoid dendritic cells (*p* = 0.001). Gender differences showed males had more intermediate monocytes (*p* = 0.009) compared to females. In summary, this study provides normal values for hematopoietic cells, highlighting age- and gender-related disparities critical for understanding hematopoietic dynamics.

## 1. Introduction

Bone marrow (BM) serves as primary site for hematopoiesis, the tightly regulated process responsible for generating and differentiating diverse hematopoietic cell lineages [1,2,3,4,5,6]. The BM of healthy individuals maintains a sufficient reservoir of precursor cells to ensure the sustained generation of hematopoietic cell lineages necessary for lifelong hematological homeostasis [1,2,3,4,5,7].

During the normal course of hematopoiesis, genetic abnormalities arising in hematopoietic cells can lead to clonal alterations, potentially disrupting the regulation of cell proliferation and differentiation [1,3,4,6,8,9]. These alterations may lead to hematopoietic neoplasms, such as leukemias, lymphomas, myeloproliferative neoplasms, and other malignant hematological diseases [1,3,5,6,8,9,10,11,12,13,14].

Flow cytometric (FC) analysis of hematopoietic cells is an essential technique in the diagnosis and monitoring of hematopoietic neoplasms in clinical settings. The most common immunophenotypic abnormalities in these cells involve alterations in antigen expression patterns relative to their healthy counterparts [1,4,6,8,15].

Understanding the expression patterns of antigens associated with specific cell lineages and maturation stages is crucial for identifying normal maturation heterogeneity and variations due to population-specific factors, aging, or gender and detecting pathological alterations [4,5]. In clinical practice, monoclonal antibodies play a key role in characterizing tissues and tumors based on antigenic expression, aiding in determining cellular or tissue origin, in accordance with the World Health Organization (WHO) criteria for hematological neoplasms [4,7,16,17,18,19].

Recent advancements in FC technology and analytical software have enhanced the ability to investigate differentiation patterns in normal bone marrow [3,9,14,15]. However, studies assessing immunophenotypic normal values in normal bone marrow hematopoietic cells remain limited [3,5,14,15,18]. Notably, most studies on normal bone normal values have relied on routine diagnostic samples specimens lacking apparent immunophenotypic abnormalities but obtained during clinical evaluation for hematological disorders [3,9,15,18].

In this study, we characterized normal gender- and age-related variations in the expression of multiple cell surface markers and maturation trajectories in bone marrow cells from healthy individuals. Using quantitative FC analysis with a 10-color methodology, we assessed marker expression across diverse hematopoietic populations, including lymphoid, myeloid and dendritic cells. Additionally, we established normal values and documented their variations, providing a valuable resource for studies focused on human BM.

## 2. Materials and Methods

### 2.1. Research Participants and Sample Obtainment

The bone marrow samples were obtained by iliac crest aspiration from 56 individuals (25 females and 31 males) undergoing orthopedic surgery, with a median age of 41 years (range: 18–91) and representing three ethnicities (29 White, 19 Mixed-race, 8 Black). Samples were collected in tubes containing ethylenediamine tetraacetic acid potassium (EDTA) tubes. All samples were from healthy individuals over 18 years old, without clinical follow-up due to hematological disease and without treatment with growth factors, chemotherapy, cytotoxic or immunosuppressive drugs, they also referred no other comorbidities. Written informed consent was obtained, and this study was approved by the Research Ethics Committee of Hospital Israelita Albert Einstein under Certificate of Ethical Review Presentation (CAAE) number 82751318.2.0000.0071, following ethical guidelines, regulations, and the Declaration of Helsinki. Details on procedures and participant characteristics are provided in Table 1 and Supplementary Data (Table S1).

### 2.2. Flow Cytometry Protocol for Hematopoietic and Immune Cell Analysis

Bone marrow samples were processed within 48 h of aspiration following Clinical and Laboratorial Standard Institute (CLSI) guidelines [20]. Automated cell counts were performed using the XN10 analyzer Sysmex^®^ (Kobe, Japan), and cell viability was assessed via 7-AAD staining.

Distinct flow cytometry panels identified immune and hematopoietic populations: B and T lymphoid cells (tubes 1 and 7), granulocytic, monocytic, and erythroid maturation (tubes 2–4), basophils, dendritic cells, and plasma cells (tube 5), and plasma cell clonality (tube 6). with antibodies and panels detailed in Supplementary Tables S2 and S3. All antibodies were titrated to minimize nonspecific staining and optimize signal-to-noise ratios.

Bone marrow samples (100 µL per tube) were stained, incubated at and using 2 mL of EXBIO^®^ (Vestec, Czech Republic) ammonium chloride (NH4Cl) solution to preserve nucleated erythrocytes. A ‘lyse-no-wash’ was used in tubes 1–5 and 7 to minimize cell loss. Flow-Count™ beads Beckman Coulter^®^ (Brea, CA, USA) ensured accurate enumeration. For intracytoplasmic Kappa and Lambda staining (tube 7), we used a labeling kit Nordic MUbio^®^ (Susteren, The Netherlands).

Data acquisition on a Navios Flow Cytometer (Beckman Coulter^®^) included ~500,000 events within the total cellularity region, including erythroblasts. Compensation was performed biannually or after equipment maintenance, following manufacturer guidelines. Daily verification of optical alignment and fluidics was conducted using Flow-Check™ Pro and Flow-Set Pro Fluorospheres (Beckman Coulter^®^).

### 2.3. Data Analysis and Gating Strategy

Data analysis was conducted using Kaluza software (version 2.3) (Beckman Coulter^®^), with standardized protocols for each tube to ensure consistency. Doublets were excluded via biparametric plots (FSC-H vs. FSC-A), followed by FSC vs. SSC gating using low thresholds to exclude cellular debris, platelets, and lipid globules. CD45 vs. SSC present in all tubes was used to refine populations of interest.

Immature and mature B cells, erythroid cells, monocytes, and granulocytes were analyzed, while T and NK cells were only identified due to maturation occurring predominantly in secondary lymphoid organs. Given the limited research on lymphoid populations in bone marrow [21,22,23,24], this study aimed to establish baseline levels in healthy individuals, considering their low frequency.

Analysis strategies were tailored to each population, with methodologies detailed in the Supplementary Data (Figures S1–S9). Quantification beads enable absolute cellularity calculations, and population identification is summarized in Supplementary Table S4. Sample hemodilution was assessed using the method by Pont et al. [9], which evaluates the ratio of immature granulocytes to neutrophils, classifying purity as high (>1.2), moderate (0.5–1.2), or low (<0.5).

### 2.4. Statistical Analysis

Quantitative variables within the groups were described using medians, 1st and 3rd quartiles, and minimum and maximum values. This approach was chosen due to the non-normal distribution of the data, verified through histograms, boxplots, quantile–quantile comparisons, and Shapiro–Wilk normality tests. Group comparisons were performed using Mann–Whitney or Kruskal–Wallis tests, depending on the number of groups, and are presented in tables. Statistical analyses were performed in SPSS (version 29.0.0) with the significance level set at α = 1%.

For both single-platform and dual-platform measurements, agreement was assessed using the intraclass correlation coefficient. For the correlation and aging groups graphs comparisons, the analysis was performed using GraphPad Prism Version 10.2.2. For the aging groups, since the data distribution was non-normal, comparisons were conducted using the Kruskal–Wallis test followed by Dunn’s post hoc test for multiple comparisons. For this analysis, the significance level was set at α = 5%.

## 3. Results

### 3.1. Concordance of Single vs. Dual Platform Measures, Bone Marrow Purity Assessment, and Dysplasia Analysis

Concordance analysis validated absolute cell counts from flow cytometry using quantification beads against measurements from the XN10 (Sysmex^®^) automated cell counter, demonstrating strong agreement for total nucleated cells, granulocytes, and erythroblasts, with robust concordance for lymphocytes and monocytes (Figure S10). Bone marrow hemodilution assessment classified 61% of samples (*n* = 34) as high purity, 36% (*n* = 20) as moderate purity, and 3% (*n* = 2) as low purity. Beyond cellular classification, dysplasia was evaluated using the Ogata score [25], revealing significant differences in the CD45 fluorescence intensity ratio between myeloblasts and lymphocytes (*p* = 0.001) and the percentage of myeloblasts (*p* = 0.005), as detailed in the Supplementary Data (Table S5).

### 3.2. Age-Related Characterization of Hematopoietic Populations and Maturation Dynamics

Hematopoietic populations were analyzed using absolute counts and percentages, stratified into three age groups [<40 years (*n* = 28), 40–60 years (*n* = 13), >60 years (*n* = 15)] to assess age-related variations. Comparative analysis confirmed previous findings and provided additional insights into cell expression patterns across the lifespan.

### 3.3. Age-Associated Variations in Bone Marrow Cellularity

Total nucleated cell counts showed significant differences across age groups (*p* = 0.001), revealing a decline in bone marrow cellularity with age (Table S6).

### 3.4. B Cell Maturation and Plasma Cell Distribution Across Age Groups

**Figure 1:**
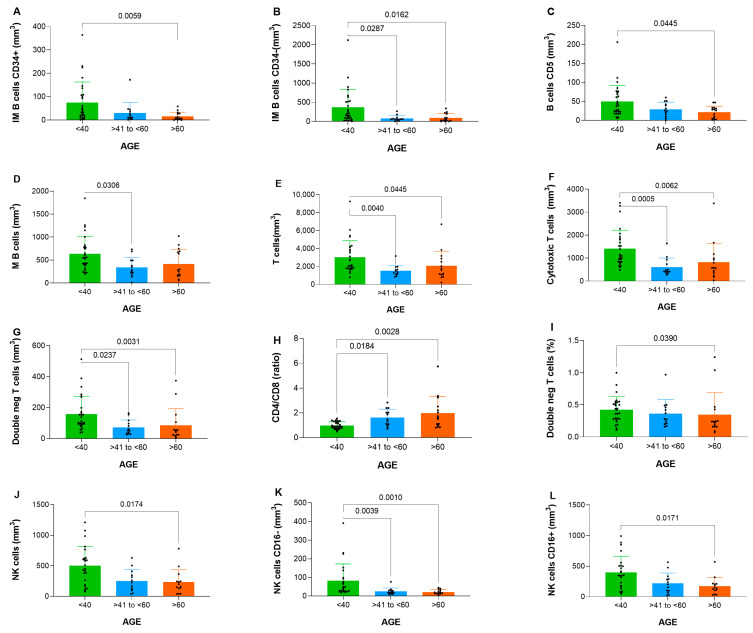
Analysis demonstrating differences for B, T and NK cells between groups (<40, >40 to <60 and >60 years old) displayed in comparatives graphs for (**A**) immature B cells CD34-positive, (**B**) immature B cells CD34-negative, (**C**) B cells CD5, (**D**) mature B cells, (**E**) T cells, (**F**) cytotoxic T cells, (**G**) double negative T cells, (**H**) CD4/CD8 ratio, (**I**) double negative T cells %, (**J**) NK cells, (**K**) NK cells CD16-negative and (**L**) NK cells CD16-positive.

Significant differences in B cell maturation were observed in absolute counts among groups for various subsets, including CD34^+^ immature B cells (*p* = 0.006) and CD34^−^ immature B cells (*p* = 0.004) (Table 2). Pairwise comparisons indicated a decline in immature B cells with aging, as illustrated in Figure 1A–D. Specifically, CD34-positive immature B cells showed significant differences between the <40 and >60 groups (*p* = 0.0059), while CD34-negative immature B cells differed between <40 and >60 groups as well as <40 and 41–60 groups (*p* = 0.0162 and 0.0287, respectively). CD5-positive B cells exhibited significant variation between <40 and >60 groups (*p* = 0.0445), whereas mature B cells differed between the <40 and 41–60 groups (*p* = 0.0306).

### 3.5. T and NK Cell Subpopulation Distribution Across Age Groups

T cell subsets analysis revealed significant variations in total T cells (*p* = 0.002), cytotoxic T cells (*p* < 0.001), and double-negative T cells (*p* = 0.0001), and the CD4/CD8 ratio (*p* = 0.001) summarized in Table 2.

Specifically, total T cells showed significant variation between <40 and >60 groups (*p* = 0.0445) and the <40 vs. 41–60 groups (*p* = 0.0040). Cytotoxic T cells differed significantly between <40 and >60 groups (*p* = 0.0062) and the <40 vs. 41–60 groups (*p* = 0.0005). Double-negative T cells also exhibited differences between <40 vs. >60 groups (*p* = 0.0031) and the <40 vs. 41–60 groups (*p* = 0.0237). The CD4/CD8 ratio varied significantly between <40 vs. >60 groups (*p* = 0.0028) and <40 vs. 41–60 groups (*p* = 0.0184). Additionally, the proportion of double-negative T cells showed a significant difference between <40 and >60 groups (*p* = 0.0390). These results highlight pronounced age-related alterations in T cell compartment. Pairwise comparisons showed significant reductions in cytotoxic T cells and double-negative T cells in elderly groups (Figure 1E–I).

NK cell subsets also varied significantly, including total NK cells (*p* = 0.007) and CD16^−^ NK cells (*p* < 0.001) (Table 2). Pairwise analysis confirmed an age-related decline in CD16^−^ NK cells (Figure 1J–L).

Specifically, total NK cells showed differences between <40 and >60 groups (*p* = 0.0174). CD16-negative NK cells exhibited significant variation between <40 and >60 groups (*p* = 0.0010) as well as between <40 and 41–60 groups (*p* = 0.0039). CD16-positive NK cells were significantly different between <40 and >60 groups (*p* = 0.0171).

### 3.6. Granulocyte Maturation and Age-Related Differences

Significant differences in granulocyte subpopulations were noted, including metamyelocytes (*p* = 0.002), neutrophils (*p* = 0.001), and basophils (*p* = 0.009) (Table 3). Mast cells (*p* = 0.002) also showed percentage differences across groups.

**Figure 2:**
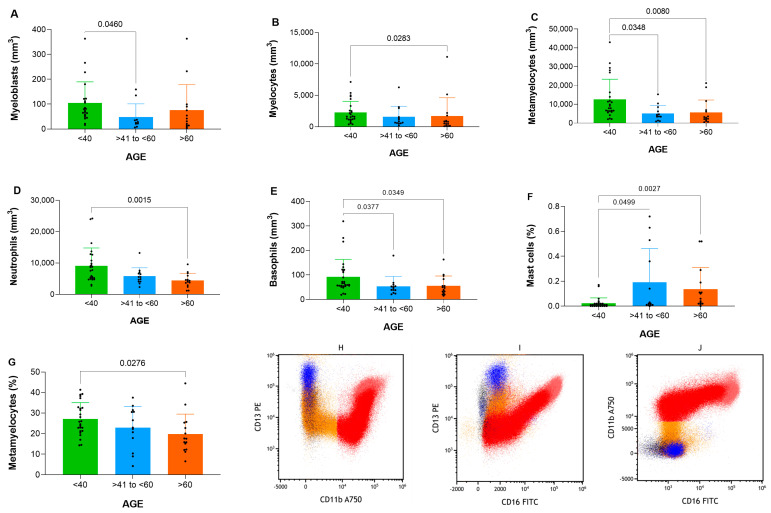
Analysis demonstrating differences for granulocytes between groups (<40, >40 to <60 and >60 years old) displayed in comparatives graphs for (**A**) myeloblast, (**B**) myelocyte, (**C**) metamyelocyte, (**D**) neutrophil, (**E**) basophil, (**F**) mast cell %, (**G**) metamyelocyte % and (**H**–**J**) granulocyte maturation curve dot plots.

Specifically, absolute numbers differed significantly for myeloblasts between the <40 and 41–60 groups (*p* = 0.0460), while myelocytes showed differences between the <40 and >60 groups (*p* = 0.0283). Metamyelocytes demonstrated variations between the <40 and >60 groups (*p* = 0.0080) and the <40 and 41–60 groups (*p* = 0.0348). Neutrophils exhibited significant differences between the <40 and >60 groups (*p* = 0.0015), whereas basophils showed variations between the <40 and >60 groups (*p* = 0.0349) and the <40 and 41–60 groups (*p* = 0.0377), as illustrated in Figure 2A–E.

In terms of percentages, mast cells displayed significant differences between the <40 and >60 groups (*p* = 0.0027) as well as <40 and 41–60 groups (*p* = 0.0499). Metamyelocytes showed variation in percentages between <40 and >60 groups (*p* = 0.0276), as depicted in Figure 2F–G.

Despite these differences in absolute and relative values, the maturation curves for the neutrophilic lineage remained consistent across all groups, following patterns characteristic of normal physiological behavior, as demonstrated in Figure 2H–J. This suggests that the overall maturation dynamics were preserved despite age-related differences and decline in cell numbers.

### 3.7. Monocytic Maturation, Dendritic Cell Subpopulations and Age-Related Differences

The evaluation of monocytic maturation revealed significant differences in absolute counts among groups for promonocytes (*p* = 0.001), mature monocytes (*p* = 0.007), classical monocytes (*p* = 0.005), and myeloid dendritic cells (*p* = 0.001), as summarized in Table 4.

**Figure 3:**
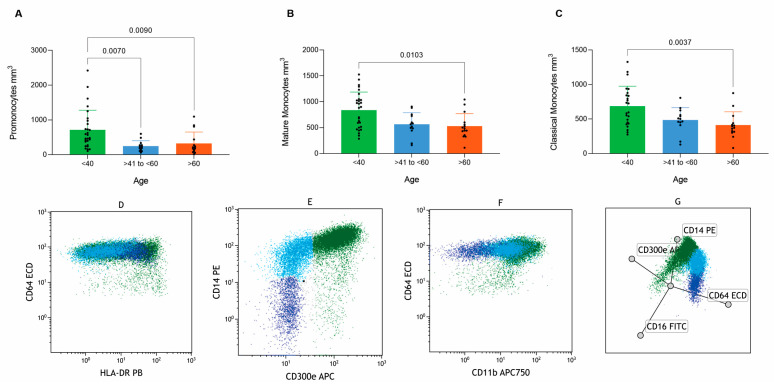
Analysis demonstrating differences for monocytes between groups (<40, >40 to <60 and >60 years old) displayed in comparatives graphs for (**A**) Promonocyte, (**B**) Mature monocyte, (**C**) Classical monocyte, and (**D**–**F**) Monocyte maturation curve dot plots, and (**G**) monocyte maturation curve displayed in the radar dot plot.

Specifically, absolute numbers differ between <40 and >60 groups as well as <40 and 41–60 groups for promonocytes (*p* = 0.0090 and *p* = 0.0070, respectively), as illustrated in Figure 3A–C.

The maturation curves of the monocytic lineage displayed consistent patterns characteristic of normal physiological behavior across all groups, as demonstrated in Figure 3D–F, complemented by radar plot analyses (Figure 3G). Although absolute values varied significantly among groups, no evident differences in the overall maturation curve dynamics were observed.

### 3.8. Erythroid Lineage Maturation and Age-Related Differences

**Figure 4:**
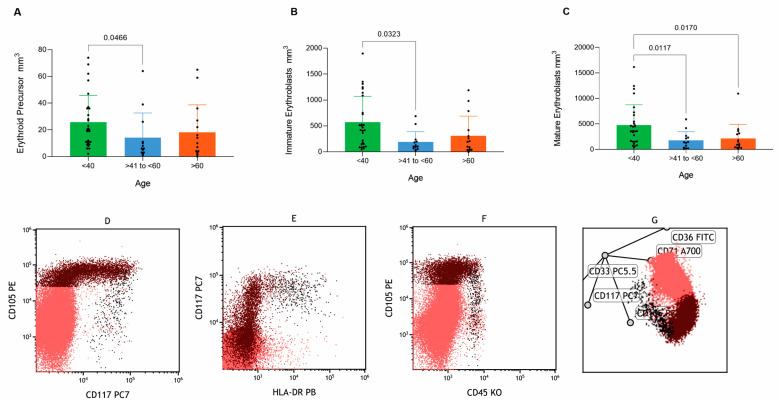
Analysis demonstrated differences for erythroid population between groups (<40, >40 to <60 and >60 years old) displayed in comparatives graphs for (**A**) Erythroid Precursor, (**B**) Immature Erythroblast, (**C**) Mature Erythroblast, (**D**–**F**) Erythroid maturation curve plots, and (**G**) Erythroid maturation curve displayed in the radar dot plot.

The analysis of erythroid maturation revealed significant differences in absolute counts among groups for mature erythroblasts (*p* = 0.002), as detailed in Table S7. Pairwise comparisons indicate significant variations in absolute numbers between <40 and 41–60 groups for erythroid precursors (*p* = 0.0466) and immature erythroblasts (*p* = 0.0323). Additionally, mature erythroblasts exhibited significant differences between <40 and >60 groups (*p* = 0.0170) as well as <40 and 41–60 groups (*p* = 0.0117), as illustrated in Figure 4A–C.

Despite these differences in absolute counts, the maturation curves of the erythroid lineage displayed consistent characteristics indicative of normal developmental patterns across all groups, as shown in Figure 4D–F and corroborated by radar plot analysis (Figure 4G). While variations in absolute counts were observed, no discernible alterations in the overall maturation curve dynamics were detected.

### 3.9. Gender-Based Analysis of Immune Cell Subpopulations

Quantitative assessments of immune cell subsets, stratified by gender, revealed significant differences in absolute counts, with higher values in males for intermediate monocytes (*p* = 0.009), as detailed in Table S8.

The gender-based analysis did not reveal significant differences in most of the cell populations studied. Despite these differences, most cell populations showed no significant gender-based variations, and no correlation between gender and age group was established.

### 3.10. Evaluation of the Kappa/Lambda Ratio in B Cells

The kappa/lambda light chain ratio was assessed in Mature B cells, CD5+ B cells, Plasma cells, and CD56+ Plasma cells. Median values (1st–3rd quartile) were 1.47 (1.31–1.73) in mature B lymphocytes, 1.59 (1.40–1.96) in CD5+ B lymphocytes, 1.47 (1.20–1.67) in plasma cells, and 1.53 (1.08–2.45) in CD56+ plasma cells, indicating a balanced light chain production and ruling out monoclonal gammopathy.

As noted by Levy et al. and Marti et al., a non-pathological kappa/lambda ratio is approximately 2:1, while monoclonality is typically defined by a ratio beyond 3:1 or 0.3:1 [26,27].

## 4. Discussion

Studies using flow cytometry have established key benchmarks for hematopoietic lineage reference values and maturation stages over the past three decades. Despite analyzing a limited number of parameters (<5), these foundational studies effectively defined normal reference ranges and the maturation stages, remaining relevant today [3,4,28,29].

However, reference values for healthy bone marrow present limitations as many studies use samples from non-healthy donors or small cohorts (<10 individuals), reducing generalizability [4,12,13]. Additional constraints include low-complexity flow cytometry equipment (fewer than 10 detection channels), limited marker panels, a narrow focus on specific cell populations, and a lack of gender or age stratification [4,9,11,13]. Furthermore, many assessments rely only on percentage-based metrics without comprehensive quantitative analyses [3,4,5,9,11,13].

Compared to previous studies, our investigation offers notable strengths, including a robust sample size of 56 healthy donors, comprehensive characterization of multiple cell populations using panels with 10-antibody per tube, and comprehensive assessments in both relative (%) and absolute (cells/mm^3^) terms. Furthermore, age- and gender-based stratification enhances applicability by capturing population variability.

Age-based stratification revealed dynamic shifts in cell distributions across the lifespan. Aging is linked to chronic low-grade inflammation due to prolonged antigenic stimulation and immune dysregulation, contributing to reduced differentiation in B lymphoid cells and a decline in T and NK cell production, as observed in this study. These changes likely underlie age-associated lymphopenia and the progressive decline in immune competence observed in the elderly [30].

In our analysis, populations that differed only in absolute counts showed relatively stable proportional distributions. This pattern likely reflects the overall decline in bone marrow cellularity with age rather than major shifts in lineage frequency, suggesting that the relative balance of these populations is preserved despite the reduction in total cell numbers.

Beyond immune cell alterations, aging induces several changes in the bone marrow, including a decline in total cell numbers, cellular senescence, shifts in lineage differentiation, and impaired hematopoietic stem cell function. These cumulative changes not only affect immune homeostasis but also have significant clinical implications, increasing the prevalence of cytopenias, such as anemia and lymphopenia, and elevating the risk of hematologic malignancies [30,31].

To validate these study findings, we compared them with existing studies, observing notable similarities. Despite using a broad age range (18 to 70 years), Pont et al. reported comparable percentage values for monocytes (4.4%), myeloblasts (0.7%), immature granulocytes (44.2%), neutrophils (22.8%), eosinophils (2.3%), basophils (0.3%), erythroblasts (12.5%), immature B cells (0.9%), mature B cells (1.8%), T cells (8.1%), NK cells (1.3%), and plasma cells (0.4%) [9].

Similarly, Matarraz et al. (2011) [11] using 4-color flow cytometry older technology, reported percentage values aligning with our results, including CD34+ immature B cells (0.07%), monocytes (4.0%), myeloblasts (1.0%), myelocytes/metamyelocytes (19.8%), neutrophils (33.0%), Eosinophils (3.5%), and Erythroblasts (15.0%) [11].

Roa-Higuera et al. (2010) [4] despite small sample size (five donors) and 5-color flow cytometry, provided reference values in both absolute (cells/mm^3^) and percentage terms, demonstrating agreement with our findings. Their reported values include monocytes (4.0%—323/mm^3^), promonocytes (0.7%—49/mm^3^), myeloblasts (1.4%—102/mm^3^), promyelocytes (1.3%—108/mm^3^), myelocytes (9.0%—800/mm^3^), metamyelocytes (22.0%—2272/mm^3^), neutrophils (30.0%—2272/mm^3^), Eosinophils (3.6%—150/mm^3^), basophils (0.2%—13/mm^3^), CD34+ immature B lymphocytes (0.2%—12/mm^3^), myeloid dendritic cells (0.3%—37/mm^3^), plasmacytoid dendritic cells (0.3%—37/mm^3^), and mast cells (0.01%—8/mm^3^) [4].

However, discrepancies were observed in some surveys, such as Brooimans et al. (2008) with granulocytes at 73.7% [5], Nies et al. (2018) with erythroblasts at 21.6% [13], and Roa-Higuera et al. (2010) with myelocytes at 9.0% (800/mm^3^) [4]. These variations may stem from differences in sample size, the inclusion of healthy bone marrow donors, and a more heterogeneous ethnic background in this study.

The Clinical and Laboratory Standards Institute (CLSI) recommends at least 120 samples to establish a reference interval [32]. However, this sample size was not reached in other studies that claimed to have defined reference values for healthy bone marrow. While our study also did not reach this threshold, it is among the few with over 50 healthy bone marrow samples, contributing valuable data to hematopoiesis research. Bone marrow collection, unlike peripheral blood sampling, is an invasive procedure requiring anesthesia. Although generally safe, it can cause discomfort, necessitate recovery time, and pose challenges in donor recruitment.

Our methodology enabled a more extensive evaluation of parameters through advanced equipment and a comprehensive monoclonal antibody panel. The inclusion of gender- and age-based stratification allowed for a more detailed interpretation of the data.

## 5. Conclusions

This study establishes absolute and relative values for key hematopoietic cell populations, including B cells, T cells, NK cells, and granulocytic, monocytic, and erythroid lineages at different maturation stages. It highlights gender- and age-related variations and heterogeneous ethnic group, offering insights into hematopoietic dynamics.

The detailed assessment of maturation stages enhances the understanding of hematopoietic development. The immunophenotypic patterns and values generated provide a robust dataset, serving as a benchmark for flow cytometry laboratories and aiding in the accurate diagnosis and monitoring of hematological malignancies and bone marrow disorders.

## Figures and Tables

**Table 1:** Clinical features.

*Healthy Donors*	*Median* *(1st and 3rd Quartiles)*	*Minimum-Maximum*
Age, years	41 (28–62)	18–91
Hemoglobin, g/dL	13.25 (11.70–14.50)	10.10–18.60
Haematocrit, %	38.25 (35.00–42.15)	28.00–56.00
Mean corpuscular volume, fL	86.45 (84.00–88.42)	79.20–98.20
Platelets, mm^3^	243.000 (200.750–286.250)	136.000–530.000

**Table 2:** Results evaluated for B, T lymphocytes and NK cells.

Cell Population		AGE			*p* Value
		<40 Years	41–60 Years	>60 Years	
Immature B-cells CD34 (positive)					
Median	(mm^3^)	37 (14–107)	12 (7–35)	7 (3–28)	**0.006**
	(%)	0.14 (0.06–0.20)	0.09 (0.03–0.17)	0.04 (0.02–0.10)	0.053
Min Max	(mm^3^)	1–364	1–172	0–58	
	(%)	0.00–0.95	0.00–0.73	0.00–0.27	
Immature B-cells CD34 (negative)					
Median	(mm^3^)	182 (81–523)	58 (48–71)	29 (17–198)	0.004
	(%)	0.5 (0.2–1.0)	0.3 (0.1–0.5)	0.3 (0.1–0.6)	0.062
Min Max	(mm^3^)	2–2120	0–265	0–339	
	(%)	0.0–5.5	0.0–1.1	0.0–1.6	
B-cells CD5 (positive)					
Median	(mm^3^)	38 (21–71)	26 (16–42)	26 (4–33)	0.043
	(%)	0.11 (0.05–0.22)	0.22 (0.06–0.23)	0.07 (0.03–0.15)	0.288
Min Max	(mm^3^)	5–206	0–60	1–47	
	(%)	0.02–0.29	0.00–0.37	0.01–0.55	
Mature B-cells					
Median	(mm^3^)	552 (394–787)	285 (220–492)	283 (162–690)	0.015
	(%)	1.7 (1.2–2.2)	1.7 (1.0–3.1)	2.1 (0.9–2.9)	0.740
Min Max	(mm^3^)	199–1844	10–734	58–1021	
	(%)	0.7–4.6	0.1–6.0	0.2–5.6	
Plasma cells (polyclonal)					
Median	(mm^3^)	49 (32–84)	35 (13–54)	42 (15–119)	0.270
	(%)	0.16 (0.07–0.24)	0.14 (0.12–0.22)	0.21 (0.13–0.44)	0.315
Min Max	(mm^3^)	1–355	2–203	1–571	
	(%)	0.00–0.82	0.01–0.69	0.01–0.74	
Plasma cells CD19 (polyclonal)					
Median	(mm^3^)	41 (25–71)	20 (9–37)	30 (11–100)	0.104
	(%)	0.13 (0.06–0.20)	0.11 (0.05–0.17)	0.15 (0.10–0.37)	0.392
Min Max	(mm^3^)	1–306	2–186	1–456	
	(%)	0.00–0.74	0.01–0.63	0.01–0.59	
Plasma cells CD56 (polyclonal)					
Median	(mm^3^)	3 (1–7)	3 (1–4)	4 (1–11)	0.780
	(%)	0.01 (0.00–0.03)	0.02 (0.01–0.02)	0.02 (0.01–0.05)	0.080
Min Max	(mm^3^)	0–34	0–9	0–93	
	(%)	0.00–0.05	0.00–0.03	0.00–0.12	
T-cells					
Median	(mm^3^)	2125 (1800–4002)	1379 (1170–1668)	1596 (1088–2876)	**0.002**
	(%)	7.4 (5.7–9.6)	8.0 (7.3–9.9)	9.4 (11.3–5.8)	0.485
Min Max	(mm^3^)	801–9251	850–3160	211–6708	
	(%)	3.6–18.0	3.4–14.1	3.3–21.4	
Helper T-cells					
Median	(mm^3^)	1031 (847–1711)	799 (542–916)	936 (557–1721)	0.090
	(%)	3.3 (2.2–4.7)	4.9 (3.2–5.7)	5.3 (3.4–6.8)	0.074
Min Max	(mm^3^)	234–5240	526–1340	172–2849	
	(%)	1.1–9.3	1.6–7.0	1.9–11.9	
Cytotoxic T-cells					
Median	(mm^3^)	1087 (840–1827)	419 (407–738)	571 (339–995)	**<0.001**
	(%)	3.4 (2.9–4.1)	2.8 (1.9–3.3)	2.7 (2.1–4.2)	0.127
Min Max	(mm^3^)	483–3405	269–1643	30–3381	
	(%)	1.7–7.5	1.2–7.0	0.5–7.9	
Double-positive T-cells					
Median	(mm^3^)	44 (29–79)	21 (9–55)	57 (20–83)	0.149
	(%)	0.12 (0.08–0.22)	0.17 (0.05–0.24)	0.21 (0.13–0.41)	0.132
Min Max	(mm^3^)	4–532	2–137	3–130	
	(%)	0.02–1.48	0.01–0.80	0.04–0.59	
Double-negative T-cells					
Median	(mm^3^)	108 (89–186)	52 (34–97)	48 (24–106)	**0.001**
	(%)	0.4 (0.3–0.5)	0.3 (0.2–0.5)	0.2 (0.4–0.2)	0.041
Min Max	(mm^3^)	39–513	25–165	5–375	
	(%)	0.1–1.0	0.2–1.0	0.1–1.2	
Ratio CD4/CD8					
Median	ratio	0.9 (0.8–1.3)	1.5 (1.1–2.0)	1.6 (1.0–2.5)	**0.001**
Min Max		0.5–1.5	0.7–2.8	0.8–5.7	
NK-cells					
Median	(mm^3^)	481 (226–626)	226 (111–345)	179 (129–246)	**0.007**
	(%)	1.1 (0.7–1.8)	1.1 (0.6–1.3)	0.9 (0.4–1.6)	0.592
Min Max	(mm^3^)	77–1210	37–631	39–781	
	(%)	0.2–5.4	0.4–4.4	0.3–3.8	
NK-cells CD16 (negative)					
Median	(mm^3^)	47 (28–96)	19 (17–30)	20 (13–30)	**<0.001**
	(%)	0.14 (0.09–0.24)	0.12 (0.07–0.19)	0.10 (0.09–0.13)	0.147
Min Max	(mm^3^)	19–392	9–77	4–45	
	(%)	0.07–0.67	0.03–0.25	0.03–0.21	
NK-cells CD16 (positive)					
Median	(mm^3^)	353 (171–507)	203 (96–289)	142 (106–210)	0.011
	(%)	0.95 (0.51–1.53)	0.93 (0.45–1.00)	0.82 (0.32–1.41)	0.784
Min Max	(mm^3^)	48–992	20–565	28–570	
	(%)	0.15–4.97	0.20–4.14	0.21–2.80	

**Table 3:** Results evaluated for granulocytic maturation.

Cell Population		AGE			*p* Value
		<40 Years	41–60 Years	>60 Years	
Myeloblasts					
Median	(mm^3^)	77 (54–121)	28 (21–37)	37 (14–90)	0.030
	(%)	0.23 (0.19–0.32)	0.14 (0.13–0.32)	0.21 (0.12–0.31)	0.726
Min Max	(mm^3^)	16–363	6–159	2–363	
	(%)	0.06–0.44	0.03–0.44	0.04–0.48	
Promyelocytes					
Median	(mm^3^)	220 (114–331)	91 (67–159)	91 (27–245)	0.029
	(%)	0.6 (0.4–07)	0.5 (0.4–0.7)	0.5 (0.3–0.8)	0.831
Min Max	(mm^3^)	41–747	38–348	3–1215	
	(%)	0.2–1.3	0.3–1.3	0.1–1.6	
Myelocytes					
Median	(mm^3^)	1818 (1172–3867)	987 (600–2258)	791 (156–1777)	0.015
	(%)	5.5 (4.0–6.7)	4.9 (4.0–6.8)	3.5 (1.8–6.7)	0.170
Min Max	(mm^3^)	376–7127	206–6265	79–11,114	
	(%)	2.4–12.2	1.4–29.3	1.4–14.8	
Metamyelocytes (band cell)					
Median	(mm^3^)	9554 (6426–15,143)	4184 (3418–7393)	2956 (1695–6947)	**0.002**
	(%)	26.6 (22.1–32.7)	24.7 (19.3–31.0)	17.5 (12.3–25.4)	0.016
Min Max	(mm^3^)	2052–42,968	906–15,294	464–21,284	
	(%)	14.3–41.4	4.2–37.5	6.5–44.5	
Neutrophils					
Median	(mm^3^)	7643 (5183–10,344)	5560 (4398–6845)	4315 (2932–5950)	**0.001**
	(%)	22.3 (16.8–26.3)	26.9 (22.8–30.4)	21.7 (15.2–30.7)	0.157
Min Max	(mm^3^)	2725–24,203	2331–13,200	1181–9588	
	(%)	8.9–43.1	20.4–42.6	9.5–43.5	
Eosinophils					
Median	(mm^3^)	605 (497–933)	338 (222–733)	429 (85–713)	0.030
	(%)	2.0 (1.7–2.6)	1.7 (1.2–3.4)	2.0 (1.2–3.6)	0.801
Min Max	(mm^3^)	292–3575	166–1402	58–2694	
	(%)	1.1–3.6	0.9–6.1	0.5–4.5	
Basophils					
Median	(mm^3^)	63 (55–119)	47 (38–54)	47 (26–83)	**0.009**
	(%)	0.20 (0.14–0.35)	0.24 (0.18–0.30)	0.22 (0.15–0.32)	0.507
Min Max	(mm^3^)	19; 319	21; 179	15; 163	
	(%)	0.07–0.50	0.10–0.61	0.13–0.59	
Mast cells					
Median	(mm^3^)	3 (2–7)	4 (3–52)	19 (4–32)	
	(%)	0.01 (0.00–0.02)	0.02 (0.01–0.36)	0.06 (0.02–0.19)	0.054
Min Max	(mm^3^)	0–64	0–255	0–82	**0.002**
	(%)	0.00–0.17	0.00–0.72	0.00–0.52	

**Table 4:** Results evaluated for monocytic maturation and dendritic cells.

Cell Population		AGE			*p* Value
		<40 Years	41–60 Years	>60 Years	
Monoblasts					
Median	(mm^3^)	39 (18–116)	50 (14–122)	25 (5–63)	0.126
	(%)	0.11 (0.05–0.31)	0.25 (0.08–0.78)	0.12 (0.02–0.34)	0.350
Min Max	(mm^3^)	6–280	3–299	0–127	
	(%)	0.02–0.77	0.02–1.02	0.00–0.75	
Promonocytes					
Median	(mm^3^)	526 (325–930)	222 (143–273)	207 (105–438)	**0.001**
	(%)	2 (1–2)	1 (1–1)	1 (1–2)	0.114
Min Max	(mm^3^)	107–2420	61–602	16–1095	
	(%)	1–3	1–2	0–3	
Mature monocytes					
Median	(mm^3^)	892 (1042–508)	566 (725–487)	484 (600–355)	**0.007**
	(%)	2 (3–1)	3 (4–3)	3 (4–2)	0.162
Min Max	(mm^3^)	286–1528	163–908	112–1044	
	(%)	1–6	1–6	1–8	
Classical monocytes					
Median	(mm^3^)	662 (443–861)	502 (452–558)	384 (315–440)	**0.005**
	(%)	1.9 (1.2–2.5)	2.4 (2.3–3.1)	2.1 (1.4–3.9)	0.211
Min Max	(mm^3^)	275–1328	131–807	81–876	
	(%)	0.8–5.1	0.7–5.1	1.3–5.5	
Intermediate monocytes					
Median	(mm^3^)	53 (19–63)	25 (13–44)	34 (21–62)	0.353
	(%)	0.10 (0.06–0.24)	0.17 (0.10–0.22)	0.20 (0.06–0.32)	0.540
Min Max	(mm^3^)	3–275	9–95	1–79	
	(%)	0.01–0.91	0.03–0.34	0.01–1.07	
Non-classical monocytes					
Median	(mm^3^)	29 (16–35)	15 (7–48)	18 (9–40)	0.816
	(%)	0.06 (0.05–0.11)	0.08 (0.05–0.28)	0.12 (0.02–0.16)	0.435
Min Max	(mm^3^)	0–66	1–134	1–97	
	(%)	0.0–7.6	0.1–15.2	0.3–16.2	
Myeloid Dendritic cells					
Median	(mm^3^)	69 (43–113)	32 (21–43)	22 (13–62)	**0.001**
	(%)	0.09 (0.04–0.12)	0.07 (0.02–0.10)	0.07 (0.02–0.12)	0.827
Min Max	(mm^3^)	15–314	5–74	4–206	
	(%)	0.01–0.18	0.00–0.24	0.01–0.51	
Plasmacytoid Dendritic cells					
Median	(mm^3^)	30 (9–53)	13 (7–16)	14 (3–46)	0.108
	(%)	0.20 (0.15–0.27)	0.15 (0.12–0.18)	0.16 (0.11–0.23)	0.827
Min Max	(mm^3^)	1–96	0–48	1–75	
	(%)	0.08–0.40	0.04–0.36	0.05–0.35	

## Data Availability

The original data will be available under request.

## Supplementary Materials

The following supporting information can be downloaded at https://www.mdpi.com/article/10.3390/cells14171392/s1, Supplementary figures containing: Figure S1: Analysis strategy for quantification of viable cells; Figure S2: Analysis strategy for cell quantification at absolute values by flux cytometry; Figure S3: Analysis strategy used to identify cell populations identified in Ery tube; Figure S4: Analysis strategy used to identify cell populations identified in Grans tube; Figure S5: Analysis strategy used to identify cell populations identified in Mono tube; Figure S6: Analysis strategy used to identify cell populations identified in PL II tube; Figure S7: Analysis strategy used to identify the cell populations in the Baso, DC, PL tube; Figure S8: Analysis strategy used to identify cell populations identified in Screening tube; Figure S9: Analysis strategy used to identify the cell populations identified in LT NK tube; Figure S10: Agreement between Single Platform and Dual Platform measures for flow cytometry and automatic cell counter XN10 (Sysmex^®^); Supplementary Tables containing: Table S1: Clinical features; Table S2: 10-color panel of monoclonal antibodies used for the entire study; Table S3: Antibodies, Dyes Used; Table S4: Immunophenotype of hematopoietic populations of interest; Table S5: Results evaluated for Ogata Score; Table S6: Results of total cell number; Table S7: Results evaluated for Erythroid maturation; Table S8: Results evaluated NK cells by gender.

## Abbreviations

The following abbreviations are used in this manuscript:

BM	Bone Marrow
CLSI	Clinical and Laboratorial Standard Institute
CAAE	Certificate of Ethical Review Presentation
FC	Flow Cytometry
FSC	Forward Scatter
NK	Natural Killer
SSC	Side Scatter
WHO	World Health Organization
