# Supplementary material for: Immunophenotypic Profile of Normal Hematopoietic Populations in Human Bone Marrow: Influence of Gender and Aging as a Basis for Reference Value Establishment
Source: Cells. 2025 Sep 6;14(17):1392. doi: 10.3390/cells14171392 (PMC12427959; doi:10.3390/cells14171392)
Supplement: Supplementary file 1 [file cells-14-01392-s001.zip › cells-3801827-supplementary.pdf]

SUPPLEMENTARY FIGURES AND TABLES

1- SUPPLEMENTARY FIGURES

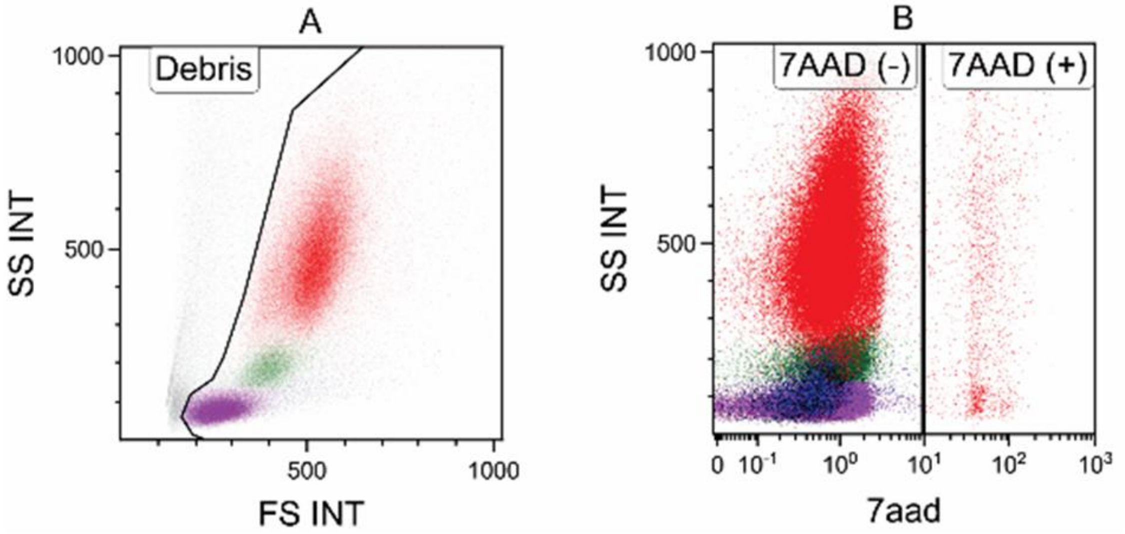

Supplementary Figure S1. Analysis strategy for quantification of viable cells. **A:** Total cell region, presented in a cross between FSC and SSC, the “debris” population was excluded **B:** In the region of total cells (without debris) two populations were classified according to the expression of the 7AAD dye, non-viable cells is“7AAD (+)” and viable cells is“7AAD (-)”.

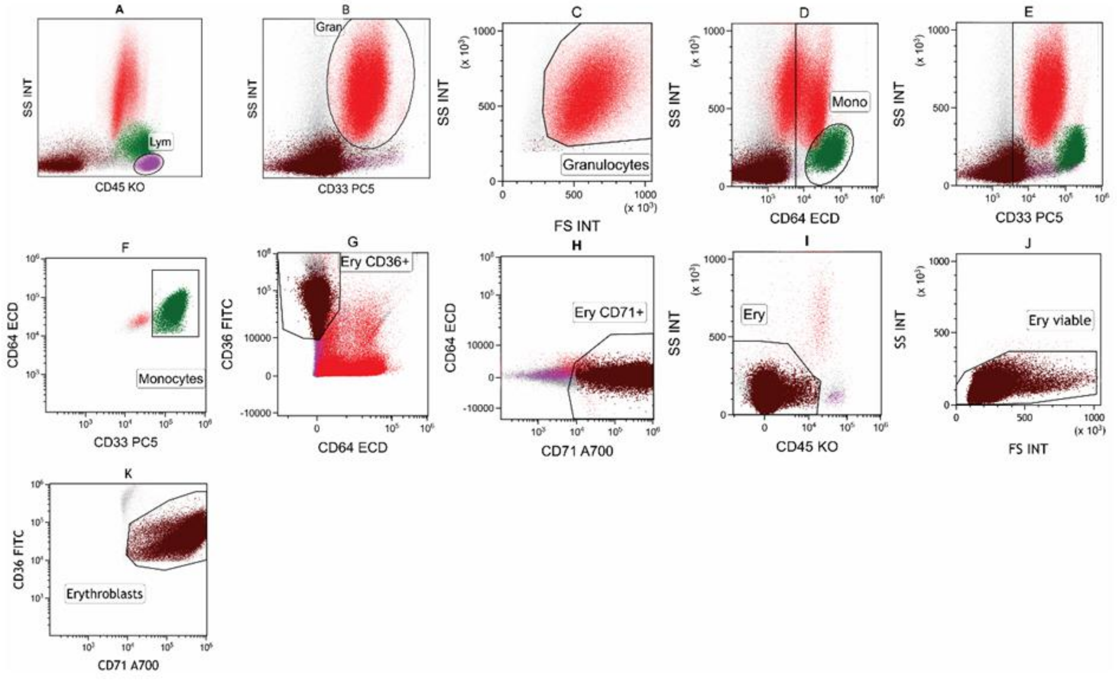

Supplementary Figure S2. Analysis strategy for cell quantification at absolute values by flow cytometry. **A:** Total nucleated cells presented in SSC and CD45

crossing, the population of low complexity cells and CD45 of strong expression and called "Lym" was selected; **B:** In the "nucleated cells" region, the population of cells of high complexity and CD33 of moderate expression and called gran region was selected; **C:** In the "Gran", presented in SSC and FSS crossing, the population of cells of high complexity and size was selected and called Granulocytes; **D:** In the "nucleated cells" region, the population of cells of low to medium complexity and CD64 of strong expression was selected and called "Mono"; **E:** In the region of total cells (without debris), presented in a crossing of SSC and CD33, it was observed whether the "Mono" strongly express CD33; **F:** In the "Mono region, the double population positive for CD64 and CD33 was selected and called "Monocytes"; **G:** In the region "nucleated cells (without monocytes)", presented in a crossing of CD36 and CD64, the population of positive CD36 and negative CD64 was selected and called "Ery CD36+"; **H:** In the "Ery CD36+" region, presented in CD64 and CD71 crossing, the population of positive CD71 and negative CD64 was selected and called "Ery CD71+"; **I:** In the region "Ery CD71+", presented in crossing of SSC and CD45, the population of low to medium complexity of CD45 from negative to weak expression and called "Ery" was selected; **J:** In the "Ery" region, presented in a SSC and FSC crossing, the population of low to medium complexity and low relative size was selected and called "Ery viable"; **K:** In the "Ery viable" region, presented in CD36 and CD71 crossing, the double population positive for CD36 and CD71 was selected and called "Erythroblasts".

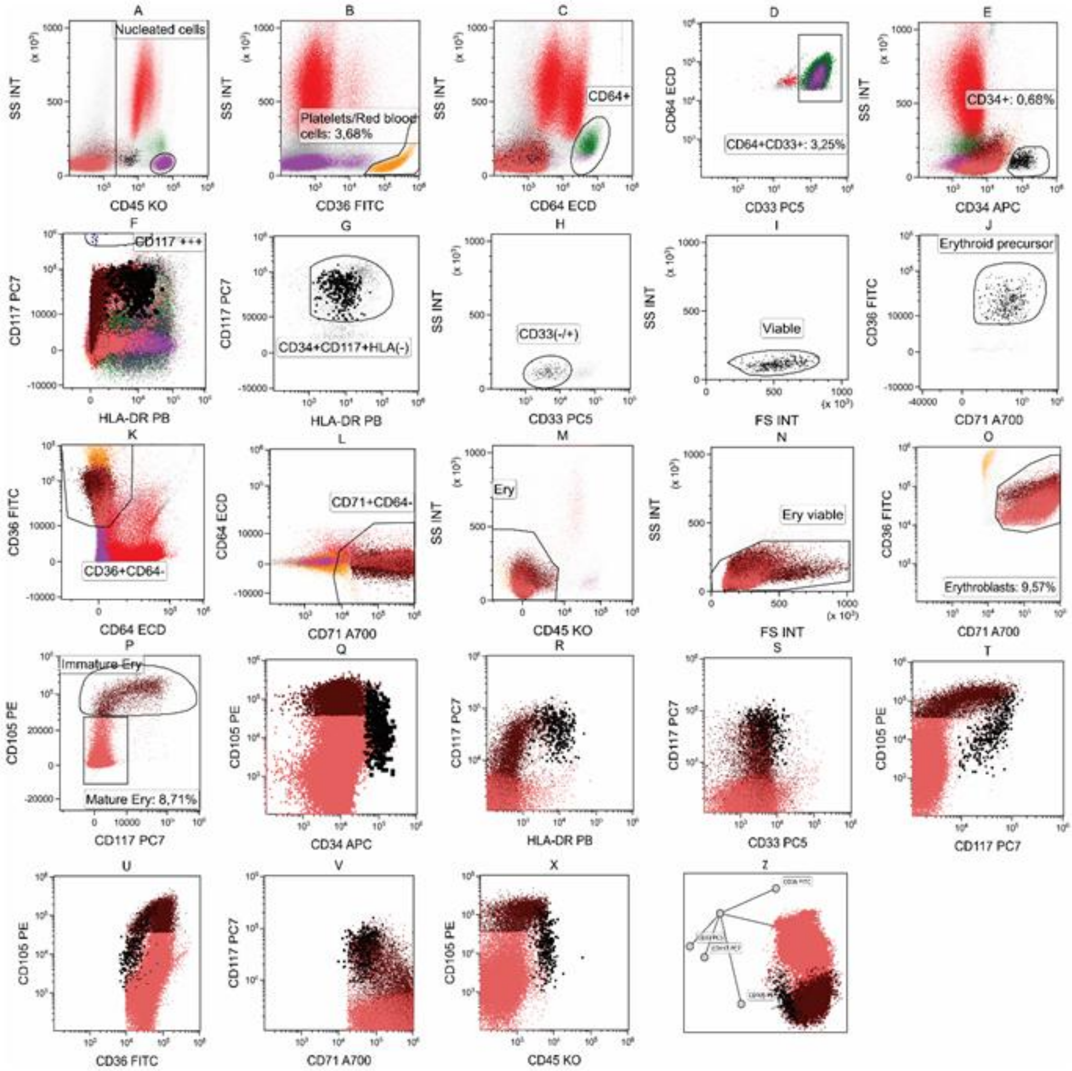

Supplementary Figure S3. Analysis strategy used to identify cell populations identified in Ery tube. **A:** Total Nucleated cells presented in SSC x CD45; **B:** In "Nucleated cells" region, the cell population expressing CD36 from moderate to strong expression was called Platelets/Red blood; **C:** In "Nucleated cells" region, the population of cells with strong CD64 expression was called CD64+; **D:** In "CD64+" region, the population of cells with strong CD33 expression was called CD64+CD33+; **E:** In "Nucleated cells" region, the population of cells with CD34 expression was called CD34+; **F:** In "Nucleated cells" region the population of cells with strong CD117 expression and was called CD117+++; **G:** In "CD34+" region, the cell population with positive expression of CD117 and negative for HLA-DR was called CD34+CD117+HLA(-); **H:** In region "CD34+CD117+HLA(-)" the low complexity population with negative to weak expression of CD33 and called CD33(-/+); **I:** In region "CD33(-/+)", the population of low to medium SSC and low FSC and called Viable; **J:** In "Viable" region, the population with positive expression for CD36 and CD71 was called an erythroid precursor; **K:** In "nucleated cells" region, the CD36 positive and CD64 negative population was called CD36+CD64-; **L:** In region "CD36+CD64-" the CD71 population was positive and CD64 negative and called "CD71+CD64-"; **M:** In region "CD71+CD64-", the population of low to medium SSC and negative CD45 to weak expression was called "Ery"; **N:** In "Ery" region, the population of low to

medium SSC and low FSC was called Ery viable; **O:** In "Ery viable" region, presented in CD36 and CD71 crossing, the double population positive for CD36 and CD71 was called Erythroblasts; **P:** In "Erythroblasts" region, the population with positive expression for CD117 and CD105 was called Immature Ery and the population with negative expression for CD117 and weak to negative for CD105 called Mature Ery. **Q-R-S-T-U-V-X:** maturation curve; **Z:** maturation curve of radar plots.

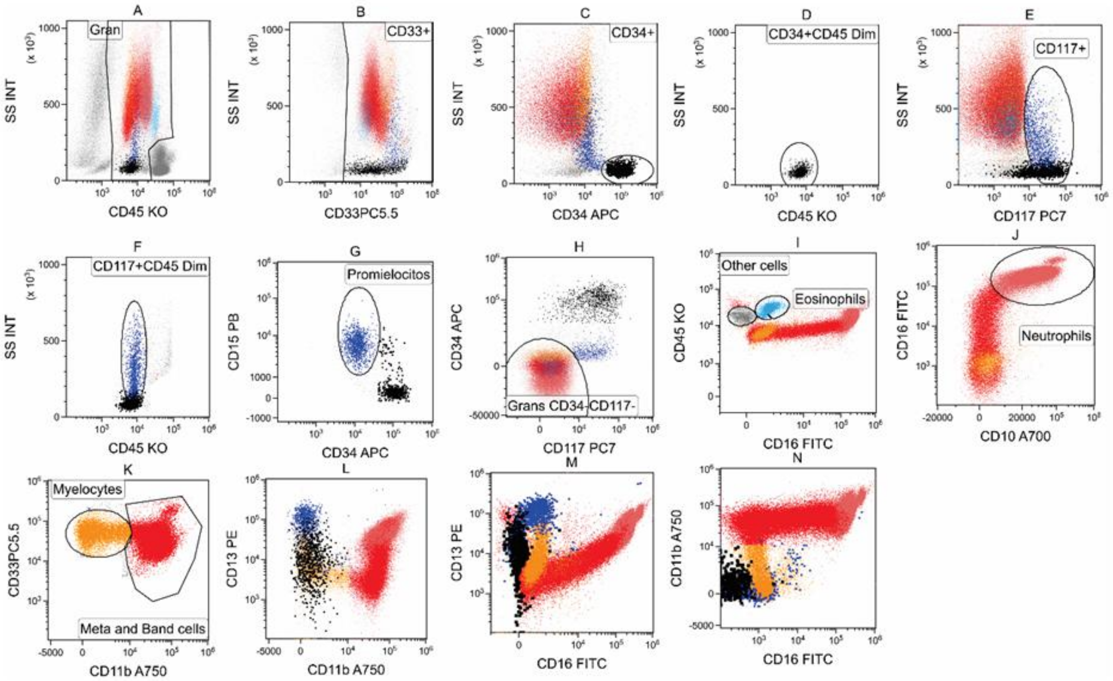

Supplementary Figure S4. Analysis strategy used to identify cell populations identified in Grans tube. **A:** Total nucleated cells presented in SSC and CD45 crossing, the population of cells from low to high complexity and CD45 from weak to moderate expression and called "Gran" was selected; **B:** In the "Gran" region, the population of cells expressing CD33 and called CD33+ region was selected; **C:** In the region "CD33, the cell population of which CD34 expresses and called CD34+ was selected; **D:** In the "CD34+" region, the population of cells of low complexity and CD45 of weak to moderate expression was selected and called CD34+CD45 dim; **E:** In the "CD33+" region, the cd117 express cell population was selected and called CD117+; **F:** In the "CD117+" region, the low to medium complexity cell population and CD45 of weak to moderate expression were selected and CD117+ CD45 dim; **G:** In the region "CD117+ CD45 dim" the population with positive expression for CD15 and negative for CD34 was selected and classified as promyelocyte; **H:** In the region "CD33+ the population with negative expression for CD34 and CD117 was selected and classified as CD34-CD117-grans; **I:** In the region "grans CD34-CD117-" the population with strong CD45 expression and negative CD16 expression and classified as Eosinophils was selected; **J:** In the region "grans CD34-CD117-" with Boolean strategy for exclusion of eosinophils, the population of cells with positive expression of CD16 and CD10 was selected and classified as Neutrophils; **K:** In the region "grans CD34-CD117-" with Boolean strategy for exclusion of Eosinophils and Neutrophils, the cell population with positive expression for CD33 and negative

for CD11b was selected and classified as Myelocytes and the population positive for CD33 and CD11b classified as Meta and Band cells. **L:** maturation curve by crossing CD13 on the Y-axis with CD11b on the X axis. **M:** maturation curve by crossing CD13 on the Y-axis with CD116 on the X axis. **N:** maturation curve by crossing CD1 1b on the Y-axis with CD16 on the X axis.

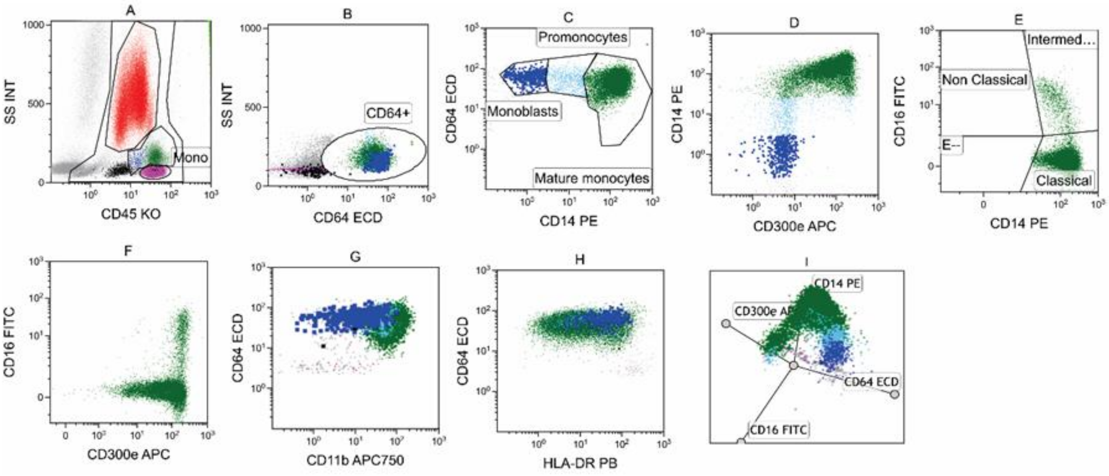

Supplementary Figure S5. Analysis strategy used to identify cell populations identified in Mono tube. **A:** Total nucleated cells presented in SSC and CD45 crossing, the population of cells of moderate complexity and CD45 moderate to strong expression was selected and called "Mono"; **B:** In the "Mono" region, the population of cells expressing CD64 and named "CD64+" was selected; **C:** In the "CD64+" region, the cell population with positive CD64 expression and absence of CD14 was classified as Monoblasts and Promonocytes and the population with positive expression paw CD64 and CD14 was classified as Matures monocytes. **D:** Mature curve by crossing CD14 on the Y-axis with CD300e on the X axis. **E:** In the "Matures monocytes" region the population with weak expression of CD14 and positive for CD16 was classified as "Non classical", the population positive for CD14 and CD16 was classified as "Intermed" and the population with positive expression for CD14 and

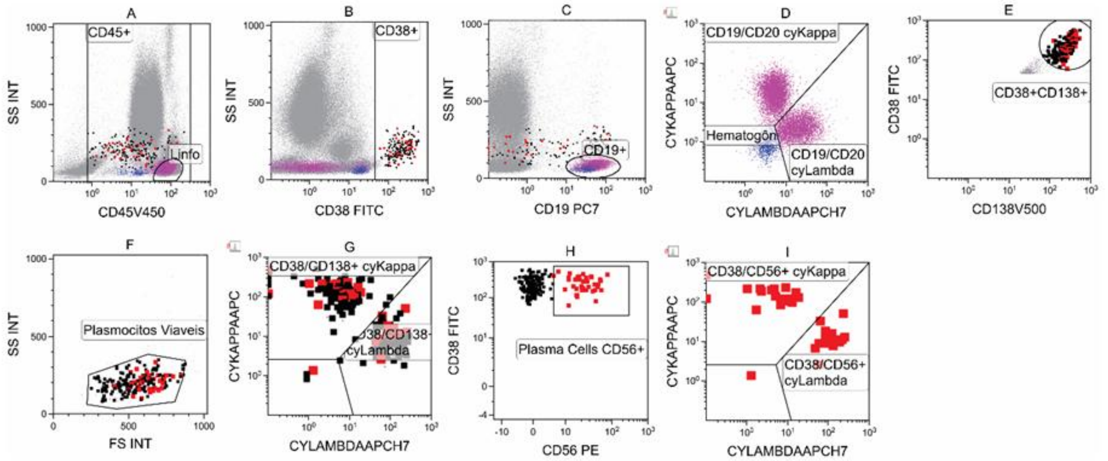

Supplementary Figure S6. Analysis strategy used to identify cell populations identified in PL II tube. **A:** Total nucleated cells presented in SSC and CD45 crossing; **B:** In the "nucleated cells" region, the population with strong positive CD38 expression was classified as CD38+; **C:** In the "nucleated cells" region, the population of cells with positive CD19 expression was classified as CD19+ (pink);

D: In the "CD19+" region, the populations were separated according to the positive expression for kappa and negative for lambda, classified as CD19/CD20 cyKappa, or positive for lambda and negative for kappa, classified as CD19/CD20 cylambda and for the double negative population (blue) it was classified as hematogonia; **E:** In the "CD38+" region, the population with positive expression of CD38 and CD138 was classified as CD38+CD138+ (black); **F:** In the "CD38+CD138+" region, the population of medium-sized and medium-complexity cells were classified as viable plasmocytes; **G:** In the "viable plasmocytes" region, the populations were separated according to the positive expression for kappa and negative for lambda, classified as CD38/CD138+ cykappa, or positive for lambda and negative for kappa, classified as CD38/CD138+ cylambda; **H:** In the "viable plasmocytes" region, the cd56-positive expression cell population was classified as Plasma Cells CD56+ (red); **I:** In the "CD56+ plasmocytes" region, the populations were separated according to the positive expression for kappa and negative for lambda, classified as CD38/CD56+ cykappa, or positive for lambda and negative for kappa, classified as CD38/CD56+ cylambda.

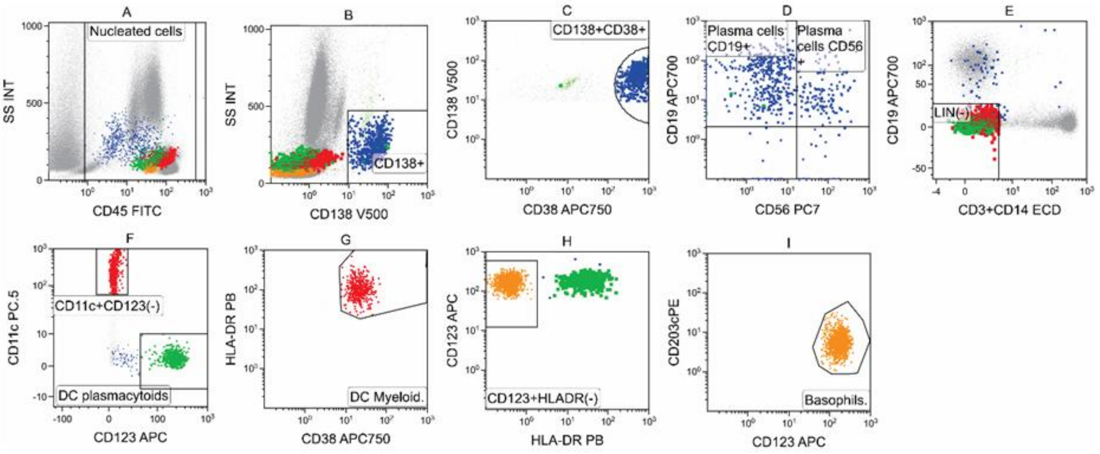

Supplementary Figure S7. Analysis strategy used to identify the cell populations in the Baso, DC, PL tube. **A:** Total Nucleated cells presented in SSC x CD45; **B:** In "Nucleated cells" region, the population with strong positive CD3/CD14 expression was called CD3+CD14+; **C:** In "Nucleated cells" region, the population of cells with positive CD11c expression was called CD11c+; **D:** In "Nucleated cells" region, the population of cells with positive CD123 expression was called CD123+; **E:** In "Nucleated cells" region, the population of cells with positive CD19 expression was called CD19+; **F:** In Nucleated cells region, the population of cells with HLA-DR positive expression was called HLA-DR+; **G:** In "Nucleated cells" region, the population of cells with positive CD138 expression was called CD138+; **H:** In "CD138+" region, the population of cells with positive expression of CD38 and CD138 was called CD138+CD38+; **I:** In region "CD138+CD38+" presented in SSC x FSC, the population of low to medium SSC and FSC was called "plasmocytes"; **J:** In "plasmocytes" region, the population of cells with positive CD19 expression was called CD138+CD19+ and the population with positive CD56 expression was called CD138+CD56+; **K:** In "Nucleated cells" region, the population with negative expression for CD3, CD14 and CD19 was called LIN(-); **L:** In region of "LIN(-) and HLA-DR+" the population with positive expression for CD11c and negative for CD123 was called CD11c+CD123- and the population with positive expression for CD123 and negative for CD11c was called DC plasmacytoid; **M:** In the region "CD11c+CD123-" the population with positive expression for HLA-DR and CD38 was called HLA-DR+CD11c+; **N:** In region "HLA-DR+CD11c+" presented in a crossing of SSC x FSC, the population of low SSC and FSC selected and was called DC myeloid; **O:** In "CD123" region, the population with positive expression for CD123 and negative for HLA-DR was called CD123+HLA-DR(-); **P:** In region "CD123+HLA-DR(-)" the population with positive expression for CD123 and CD203c was called "CD203c+CD123+"; **Q:** In region "CD203c+CD123+" presented in crossing SSC and FSC, the population of low SSC and FSC was called Basophils.

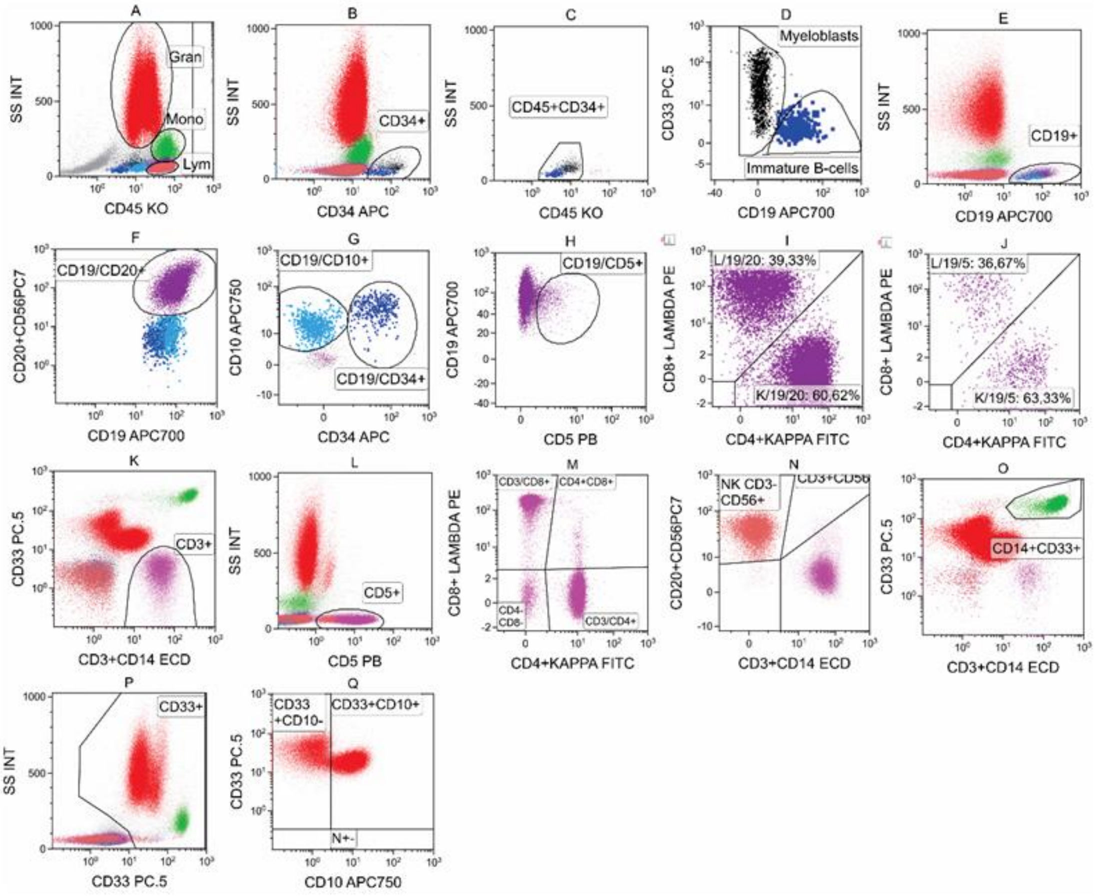

Supplementary Figure S8. Analysis strategy used to identify cell populations identified in Screening tube. **A:** Total nucleated cells; **B:** In "Nucleated cells", the population with positive CD34 expression was called CD34+; **C:** In "CD34+", the population with negative to weak CD45 expression was called CD45+CD34+; **D:** In "CD45+CD34+", according to the expression of CD19 positive with CD33 negative, was called Immature B-cells, and positive for CD33 and negative for CD19, was called Myeloblasts; **E:** In "Nucleated cells", the population with positive CD19 expression was called CD19+; **F:** In "CD19+" the population with positive CD20 expression was called CD19+CD20+; **G:** In “CD19+” the population with positive CD10 expression and negative CD34 was called CD19+CD10+ and the population with positive expression of CD10 and CD34 was called CD19+CD34+; **H:** In “CD19+”, the population with positive CD5 expression was called CD19+CD5+; **I:** In "CD19+CD20+" two populations were separated according to expression for kappa and lambda, was called “L/19/20” or “K/19/20”; **J:** In "CD19+CD5+" two populations were separated according to expression for kappa and lambda, was called “L/19/5” or “K/19/5”; **K:** In "Nucleated cells", the population with positive CD3 expression and negative for CD33 was called “CD3+”; **L:** In "Nucleated cells", the population of cells with positive CD5 expression was called “CD5+”; **M:** The "CD3+", was separated into four populations according to the expression positive for CD4 and CD8, was called “CD3/CD4+”, “CD3/CD8+”, “CD4+CD8+” or “CD4-CD8-“; **N:** In "Lym" through the initial gate of histogram A and with a Boolean strategy to exclude the “CD19+”, the population with positive expression of CD56 and negative for CD3 was called “NK CD3-CD56+”; **O:** In "Nucleated cells", the population with positive CD14 expression and negative for CD3 was called “CD14+CD33+”; **P:** In

"Nucleated cells", the population with an increase in SSC and intermediate expression of CD33 was called “CD33+”; **Q:** The "Gran" was separated into two populations according to expression for CD10, was called “CD33+CD10+” or “CD33+CD10-“.

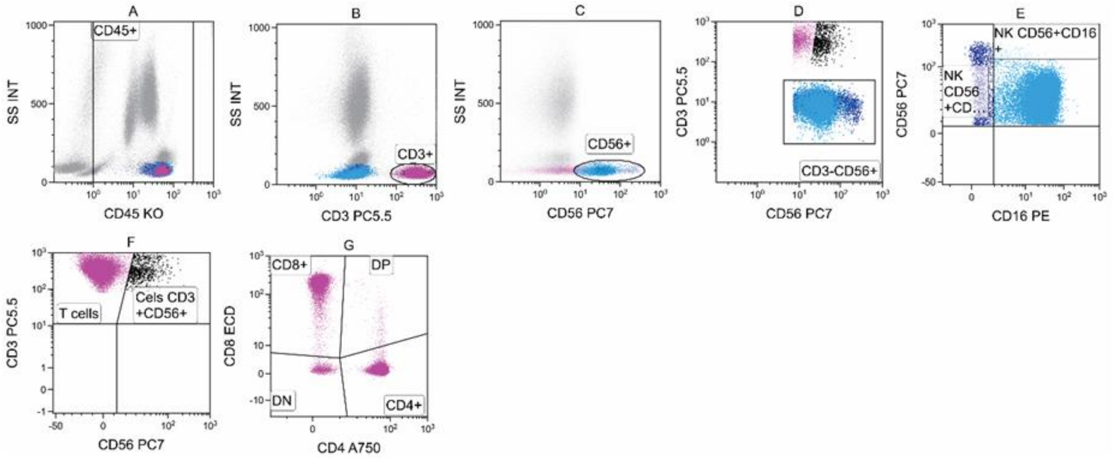

Supplementary Figure S9. Analysis strategy used to identify the cell populations identified in LT NK tube. **A:** Total nucleated cells presented in SSC and CD45 crossing; **B:** In the "nucleated cells" region, the population of cells with positive CD3 expression was classified as CD3+ (pink); **C:** In the "nucleated cells" region, the population of cells with positive CD56 expression was classified as CD56+; **D:** In the "CD56+" region, the population with positive CD56 expression and CD3 negative expression was classified as CD3 +CD56-; **E:** In the "CD3+CD56-" region, the populations were separated according to the positive expression for CD16, classified as NK CD56+CD16+ (light blue), or negative for CD16 classified as NK CD56+CD16- (dark blue); **F:** In the region of "CD3+" the population with positive CD3 expression and CD56 negative expression was classified as T cells and the population with positive CD3 expression and positive expression for CD56 was classified as CD3 +CD56+ cells (black); **G:** The "T cells" region was separated into four populations according to the expression positive for CD4 and negative for CD8 classified as auxiliary T cells, positive for CD8 and negative for CD4 classified as cytotoxic T cells, positive for CD4 and CD8 classified with double positive and negative T cells for CD4 and CD8 classified with negative double T cells.

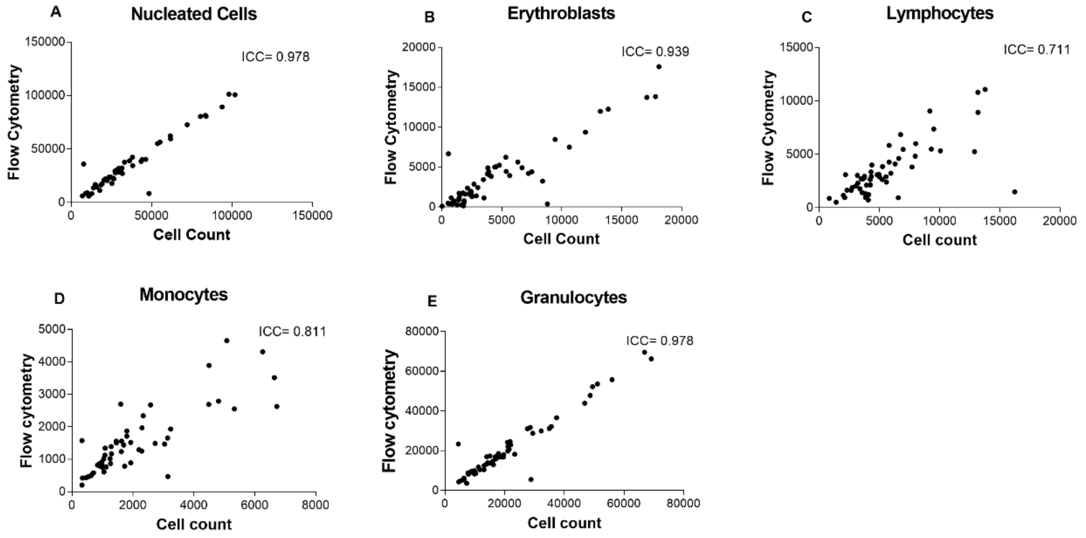

Supplementary Figure S10. Agreement between Single Platform and Dual Platform measures for flow cytometry and automatic cell counter XN10 (Sysmex®) (A) Total nucleated cells **(B)** Erythroblasts, **(C)** Lymphocytes, **(D)** Monocyte and **(E)** Granulocytes.

2-SUPPLEMENTARY TABLES

Table S1. Type of Surgery

TYPE OF SURGERY	QUANTITY
Osteosynthesis	41
Arthroplasty	5
Removal of synthesis material or	3
Pseudoarthrorse treatment	2
Tenorrhaphy	2
Ligament Reconstruction	2
Arthroscopy	1

Table S2. 10-color panel of monoclonal antibodies used for the entire study

Fluorochrome	Screening Tube 1	Granulocyte Lineage Tube 2	Monocyte Lineage Tube 3	Erythrocyte Lineage Tube 4	Basophils, DC, PL Tube 5	PL II Tube 6	LT NK Tube 7
FITC	CD4 / CLK(s)	CD16	CD16	CD36	CD45	CD38	CD57
PE	CD8 / CLL(s)	CD13	CD14	CD105	CD203c	CD56	CD16
ECD	CD3 / CD14	CD38	CD64	CD64	CD38		CD8
PC5	CD33	CD33	CD34	CD33	CD33		CD3
PC7	CD20 / CD56	CD117	CD117	CD117	CD56	CD19	CD56
APC	CD34	CD34	CD300e	CD34	CD123	CLK(i)	CD2
APC A700	CD19	CD10		CD71			CD7
APC A750	CD10	CD11b	CD11b		CD19	CLL(i)	CD4
PB	CD5	CD15	HLA-DR	HLA-DR	HLA-DR	CD45	CD5
KO	CD45	CD45	CD45	CD45	CD138	CD138	CD45

CLK: light chain Kappa; CLL: light chain lambda; (i): intracytoplasmic and (s): surface; DC: Dendritic cells; PL: Plasmocytes; LT-NK: T lymphocytes-NK cells.

Table S3. Antibodies, Dyes Used

CD/ Dye / Reagents	Fluorophores	Clone	Reference	Manufacturer
7-AAD			A07704	Beckman Coulter
CD45 FITC	FITC	J33	A07782	Beckman Coulter
CD4 FITC	FITC	13B8.2	A07750	Beckman Coulter
KAPPA FITC	FITC	Polyclonal	A64828	Beckman Coulter
CD16 FITC	FITC	3G8	IM0814U	Beckman Coulter
CD36 FITC	FITC	FA6.152	IM0766U	Beckman Coulter
CD38 FITC	FITC	T16	A07778	Beckman Coulter
CD57 FITC	FITC	NC1	IM0466U	Beckman Coulter
CD8 PE	PE	B9.11	A07757	Beckman Coulter
LAMBDA PE	PE	Polyclonal	PN64827	Beckman Coulter
CD13 PE	PE	L138	L138	BD
CD14 PE	PE	MoP9	347497	BD
CD105 PE	PE	1G2	B76299	Beckman Coulter
CD203c PE	PE	97A6	IM3575	Beckman Coulter
CD56 PE	PE	N901	IM2073U	Beckman Coulter
CD16 PE	PE	3G8	A07766	Beckman Coulter
CD3 ECD	ECD	UCHT1	A07748	Beckman Coulter
CD14 ECD	ECD	RMO52	IM2707U	Beckman Coulter
CD38 ECD	ECD	LS198-4-3	A99022	Beckman Coulter
CD64 ECD	ECD	22	A98434	Beckman Coulter
CD8 ECD	ECD	SFCI21Thy2D3	737659	Beckman Coulter
CD33 PC5.5	PC5.5	D3HL60.251	B36289	Beckman Coulter
CD34 Percp.5.5	Percp.cy.5	8G12	347203	BD

CD11c PC5.5	PC5.5	BU15	B19719	Beckman Coulter
CD3 PC5.5	PC5.5	UCHT1	A66327	Beckman Coulter
CD20 PC7	PC7	B9E9	IM3629	Beckman Coulter
CD56 PC7	PC7	N902 (NKH-1)	A21692	Beckman Coulter
CD117 PC7	PC7	104D2D1	IM3698	Beckman Coulter
CD19 PC7	PC7	J3-119	IM3628	Beckman Coulter
CD34 APC	APC	581	IM2472	Beckman Coulter
IREM-2	APC	UP-H2	656158	BD
CD123 APC	APC	SSDCLY107D2	B06376	Beckman Coulter
CD2 APC	APC	39C1.5	A60794	Beckman Coulter
KAPPA APC	APC	TB28-2	1A-674-T100	EXBIO
CD326 APC	APC	9C4	324208	Biolegend
CD19 A700	APC-A700	J3-119	A78837	Beckman Coulter
CD10 A700	APC-A700	ALB1	A86353	Beckman Coulter
CD71 A700	APC-A700	YDJ1.2.2	A97051	Beckman Coulter
CD7 A700	APC-A700	8H8.1	B36290	Beckman Coulter
CD10 A750	APC-A750	ALB1	A86353	Beckman Coulter
CD11b A750	APC-A750	Baer1	B36295	Beckman Coulter
CD38 A750	APC-A750	LS198-4-3	A86049	Beckman Coulter
CD4 A750	APC-A750	13B8.2	A94682	Beckman Coulter
Lambda	APC-H7	1-155-2	656648	BD
CD5 PB	Pacific Blue	BL1a	A82790	Beckman Coulter
CD15 PB	Pacific Blue	80H5	A74775	Beckman Coulter
HLA-DR PB	Pacific Blue	Immu-357	B36291	Beckman Coulter
CD45 V450	V450	2D1	642275	BD
CD45 KO	Krome Orange	J33	B36294	Beckman Coulter
CD138 V500	V500	MI15	650659	BD
Excellyse Live			ED706B	EXBIO
Flow Count			7547053	Beckman Coulter
PBS			70011-044	GIBCO
FIX & PERM			GAS-002-1	Nordic MUbio

Table S4. Immunophenotype of hematopoietic populations of interest

CELL-SUBTYPE	IMMUNOPHENOTYPE
Immature B-cells CD34(positive)	CD19+CD34+
Immature B-cells CD34(negative)	CD19+CD10+CD34-
B-cells CD5	CD19+CD5+
Mature B-cells	CD19+CD20+
Plasma cells CD19	CD38+CD138+CD19+
Plasma cells CD56	CD38+CD138+CD56+
T-cells	CD3+
Helper T-cells	CD3+CD4+CD8-
Cytotoxic T-cells	CD3+CD8+CD4-
Double positive T-cells	CD3+CD4+CD8+
Double negative T-cells	CD3+CD4-CD8-
NK-cells	CD56+CD3- (inside lymphocytes gate)
NK-cells CD16 (negative)	CD56+CD3-CD16-
NK-cells CD16 (positive)	CD56+CD16+CD3-
Myeloblasts	CD34+CD33+CD45+
Promyelocytes	(CD33+CD117+CD15+)
Myelocytes	(CD33+CD16-CD10-)
Metamyelocytes (band cell)	(CD33+CD11b+CD10-CD16+)
Neutrophils	CD33+CD16+CD10+
Eosinophils	CD33+SSC++CD45++CD34-CD117-CD16-
Basophils	CD123+CD203c+HLADR-
Mast cells	CD117+++SSC++
Monoblasts	CD34+CD64+CD14-
Promonocytes	CD64+CD300e-CD14-
Mature Monocytes	CD64+CD300e+CD14+
Classical Monocytes	CD14+CD16-
Intermediate Monocytes	CD14+CD16+
Non-classical Monocytes	CD14(weak)/CD16+
Myeloid Dendritic cells	CD19(-) CD3/CD14(-) CD123(-) CD11c+HLA-DR+
Plasmacytoid Dendritic cells	CD19(-) CD3/CD14(-) CD11c (-) CD123+HLA-DR+
Erythroid Precursor	CD34+CD117+CD36+CD71+
Immature Erythroblasts	CD36+CD71+CD117+CD105+
Mature Erythroblasts	CD36+CD71+

Table S5. Results evaluated for Ogata Score

Ogata Score		AGE			p Value
		<40 years	41 - 60 years	>60 years	
SSC (>6 = 1 score)					
Median	%	8.0 (7.2-8.4)	8.0 (7.3-8.4)	7.8 (7.3-8.4)	0.988
Min		5.9-10.1	6.5-9.4	6.0-11.1	
CD45 (< 4 or >7.5 = 1 score)					
Median	%	5.9 (5.5-6.4)	6.0 (5.6-6.1)	5.2 (5.1-5.5)	**0.001**
Min		4.9-8.1	4.9-7.1	4.8-6.2	
Myeloblasts (>2 = 1 score)					
Median	%	0.8 (0.6-1.0)	0.4 (0.3-0.6)	0.6 (0.4-0.8)	**0.005**
Min		0.3-1.4	0.2-1.2	0.1-1.9	
Immature B-cells (<5 = 1 score)					
Median	%	14.5 (9.7-21.1)	14.1 (9.7-26.0)	9.5 (1.7-16.5)	0.133
Min		0.4-41.5	1.4-52.3	0.2-31.3	

Table S6- Results of total cell number

Cell Population	AGE <40 years	41 -60 years	>60 years	p Value
**Nucleated cells**				
Median (mm³)	36020 (28850-57966)	20951 (15552-27874)	21952 (9224–29409)	**0.001**
Min Max (mm³)	14445 - 101237	8263 - 40373	6115 - 80440	

Table S7- Results evaluated for Erythroid maturation

Population		AGE <40 years	41 - 60 years	>60 years	p Value
**Erythroid Precursor**					
Median	(mm³)	20 (11-36)	6 (3-11)	10 (3-27)	0.028
	(%)	0.06 (0.04- 0.08)	0.05 (0.03- 0.06)	0.05 (0.03- 0.08)	0.552
Min Max	(mm³)	2- 74	1- 64	0- 65	
	(%)	0.00- 0.13	0.01- 0.20	0.00- 0.12	
**Immature Erythroblasts**					
Median	(mm³)	445 (149-920)	104 (70-199)	194 (41-419)	0.015
	(%)	1.2 (0.6-1.5)	0.6 (0.4-0.9)	0.9 (0.4-1.2)	0.146
Min Max	(mm³)	47- 1898	23- 689	1- 1189	
	(%)	0.2- 3.4	0.3- 1.9	0.0- 2.5	
**Mature Erythroblasts**					
Median	(mm³)	3606 (1559-6102)	1400 (350-2325)	1046 (402-3161)	**0.002**
	(%)	9.8 (4.2-14.9)	4.6 (4.2-10.3)	5.1 (3.9-10.8)	**0.002**
Min Max	(mm³)	380- 16153	77- 5908	93- 10947	
	(%)	2.6- 22.1	0.7- 16.5	1.5- 16.9	

Table S8 - Results evaluated NK cells by gender

Population		GENDER Females	Males	p Value
**NK-cells**				
Median	(mm³)	216 (94 - 366)	409 (169 - 604)	0.029
	(%)	0.7 (0.4 - 1.1)	1.2 (0.9 - 2.0)	**0.003**
Min Max	(mm³)	37 - 1077	42 - 1210	
	(%)	0.2 - 3.8	0.4 - 5.4	
**NK-cells CD16 (negative)**				
Median	(mm³)	23 (17 - 38)	30 (23 - 65)	0.162
	(%)	0.12 (0.08 - 0.18)	0.11 (0.09 - 0.21)	0.237
Min Max	(mm³)	11 - 232	4 - 392	
	(%)	0.03 - 0.27	0.06 - 0.67	
**NK-cells CD16 (positive)**				
Median	(mm³)	165 (71 - 319)	317 (154 - 498)	0.027
	(%)	0.44 (0.24 - 0.95)	1.00 (0.66 - 1.62)	**0.006**
Min Max	(mm³)	20 - 850	38 - 992	
	(%)	0.15 - 2.80	0.21 - 4.97	
